# In Reply: Costa et al

**DOI:** 10.1186/s13054-022-04254-z

**Published:** 2022-12-30

**Authors:** Jordan Spurling, Nicole Glowacki, Marc McDowell

**Affiliations:** 1 University of Illinois at Chicago, Chicago, USA; 2 Advocate Research Institution, Milwaukee, USA

We read with interest the article by Costa et al. reporting on coagulation factor Xa recombinant, inactivated-zhzo, which discusses optimal management strategies for the reversal of factor Xa (FXa) inhibitor-associated uncontrolled or life-threatening bleeding [1]. We commend the authors’ efforts to determine the effectiveness and safety of coagulation factor Xa recombinant, inactivated-zhzo compared to Four-Factor Prothrombin Complex Concentrate (4F-PCC) in the management of apixaban- or rivaroxaban-associated intracranial hemorrhage. After carefully reading the article, we would like to share our commentary.

Costa et al. excluded patients with a Glasgow Coma Score (GCS) less than 7. The mean GCS in the baseline demographics of this trial both before and after propensity score matching was 14. This leads the reviewer to debate the applicability of these trial results to patients presenting with more severe injuries. The exclusion of a GCS of 7 also leads the reviewer to have reservations regarding the trial’s endpoint of overall 30-day mortality rate. Patients presenting with a GCS of 14 are less likely to present with a large hematoma and therefore are expected to have less severe sequelae. Furthermore, the trauma subset included in this trial lacks details about the severity of injury that may provide insight to the reader. The trauma subset does not provide an injury severity score to frame our understanding of the injury. Without this detail, the reader is left uninformed of a significant nuance regarding baseline severity and therefore the efficacy of treatment. The relatively high GCS cutoff combined with the missing injury severity scoring in the trauma subset may lead the reader to conclude that the comparison between coagulation factor Xa recombinant, inactivated-zhzo and 4F-PCC on mortality is appropriate to apply in moderate-to-severe FXa inhibitor-associated life-threatening intracranial bleeding.

The 4F-PCC weight-based dose suggested by the Society for Neurocritical Care Society and the Society of Critical Care Medicine in 2016 recommends 50 units/kilogram (kg) to prevent hematoma expansion [2]. However, in this trial 79.3% (after propensity score matching) of the 4F-PCC group received a 25 unit/kg dose. While this study was conducted between 2015 and 2020, the authors should consider that the dose of 4F-PCC may have been inadequate. The mechanism of 4F-PCC relies on the repletion of clotting factors (II, VII, IX, X, protein C and S) to overcome the mechanism of the anticoagulant taken in association with major bleeding. At the dose of 25 units/kg tested in this trial, the 4F-PCC may not provide adequate clotting factor supplementation to achieve hemostasis. While 4F-PCC was inadequately dosed in a large percentage of their treatment arm, 96.6% of the coagulation factor Xa recombinant, inactivated-zhzo group (after propensity score matching) was dosed at 400 mg bolus followed by a 440 mg infusion as recommended by the package insert [3]. Therefore, a direct comparison of package insert recommended dosing of coagulation factor Xa recombinant, inactivated-zhzo compared with nearly 80% of the 4F-PCC group being underdosed according to the Society of Neurocritical Care Society and the Society of Critical Care Medicine may not provide insight into the superiority of one agent over the other [2, 3]. Furthermore, the timing of the last dose of apixaban or rivaroxaban is not commented on in this trial which may have helped the reader evaluate dosing in relation to replenishing clotting factors. Dosing efficacy, which is reliant of clotting factors, is directly related to active DOAC remaining at the time of the bleed which correlates with the timing of the DOAC prior to the bleed.

The statistics in this trial utilize propensity matching to match patients in coagulation factor Xa recombinant, inactivated-zhzo arm from the ANNEXA-4 trial to serve as a synthetic control arm for the patients enrolled in the 4F-PCC group during this study period [4]. Propensity score matching is a major limitation of this trial because it utilizes data sets from a previous trial and incorporates these patients into a separate trial to draw results from the collaboration. Additionally, propensity score matching appears to make baseline demographics similar; however, propensity matching lacks specificity regarding key differences between the groups. This leaves the reviewers to believe that conclusions made from the joint data sets may be limited.

We, therefore, caution that conclusions drawn about the comparison in hemostatic effectiveness and mortality in coagulation factor Xa recombinant, inactivated-zhzo versus 4F-PCC may be limited.

## Data Availability

Not applicable.

## Abbreviations

DOAC: Direct oral anticoagulant
FXa: Factor Xa
GCS: Glasgow Coma Score
Kg: Kilogram
4F-PCC: Four-Factor Prothrombin Complex Concentrate
